# High-Resolution Single-Molecule Structure Revealed by Electron Microscopy and Individual Particle Electron Tomography

**DOI:** 10.4172/2161-0398.1000e103

**Published:** 2012-05-22

**Authors:** Lei Zhang, Gang Ren

**Affiliations:** The Molecular Foundry, Lawrence Berkeley National Laboratory, Berkeley, USA

**Keywords:** Protein dynamics, Protein structure, Equilibrium fluctuation, Single-molecule structure, Individual-Particle Electron Tomography (IPET), Electron Microscopy (EM), Electron Tomography (ET), Cryo-Electron Tomography (CryoET), Optimized negative-staining (OpNS), Cryo-positive-staining (Cryo-PS)

## Abstract

The protein is naturally dynamic and heterogeneous in solution. Protein dynamics involves both equilibrium fluctuations that regulate biological function and other non-equilibrium effects of biological motors, which convert chemical energy to mechanical energy. However, a single, unique structure of protein determined from X-ray crystal and conventional single-particle electron microscopy is insufficient to encompass the dynamic nature of proteins in solution. Structure determination of dynamic and heterogeneous protein is essentially required the determination of each individual particle of protein. Recently, Drs. Gang Ren and Lei Zhan published the first single molecule three-dimensional (3D) EM images of individual proteins ever obtained with enough clarity to determine their structure, an IgG antibody (14 Å resolution) and a 17nm HDL (36 Å resolution). These results depended upon four innovations: i) improved cryo-electron microscopy (cryoEM) sample preparation and Electron microscopy (EM) operation conditions resulted in the successful imaging of a 17 nm HDL particle (120–200kDa) by cryo-electron tomography (cryoET); ii) developed an optimized NS (OpNS) protocol that eliminates the rouleau artifact that has plagued EM research for three decades. This OpNS protocol provides high-contrast single lipoprotein images with similar size (<5%) and shape (<5%) to that seen by cryoEM; iii) developed a high-resolution and high contrast sample preparation protocol, cryo-positive-staining (cryoPS) that allows direct visualization of the secondary structure of a small protein, such as the β-strands in CETP and the helical double belt of apoA-I in spherical HDL; iv) developed a robust tomography reconstruction method, Individual Particle Electron Tomography (IPET) that is a high-resolution, high throughput reconstruction method that, to the best of our knowledge, is the only method for determining an individual protein structure. Remarkably, IPET went against the conventional wisdom that a single protein can NOT be reconstructed by EM and this opens a door for the study of protein dynamics via a particle-by-particle structural comparison.

## Introduction

A single, unique structure of protein determined by X-ray crystal is often used for studies in structure–function relationships. However, the protein is naturally dynamic and fluctuation in solution, the single unique structure is insufficient to illuminate the dynamic character and “personalities” [1,2]. Other than a theoretical calculation approach, such as molecular dynamics simulation is able to link structure and dynamics by enabling the exploration of the conformational energy landscape accessible to protein molecules [1,2], there is no experimental approach to determine the dynamic structure at atomic-resolution level because current structure determination techniques, including X-ray crystallography and single-particle electron microscopy, require an averaging of the signals from thousands to millions different particles. A fundamental solution for structure determination of dynamics protein is to determine the structure of each individual particle of protein.

Electron Tomography (ET) is an experimental tool to provide different tilted viewing images of an individual targeted biological object from a series of tilted angles [3,4]. The first 3D structure of an individual protein was the negative-staining (NS) reconstruction of fatty acid synthetase molecule achieved by [3]. However, the structure was argued because the molecule received a radiation dose that exceeds the limits found by Unwin and Henderson by a large factor [5]. Since then, it has been a long time that people thought a single protein is insufficient to provide sufficient signal for a 3D reconstruction. Recently, Ren and Zhang broke this religion by reporting a first 3D image of an individual protein under the low-dose cryo-electron microscope (cryoET) and NS ET [6]. These single-molecule, 3D EM images of individual proteins ever obtained with enough clarity to determine the protein structure. The 3D reconstructions include two individual IgG antibodies [14,15] Å resolution) that has a molecular mass (MM) of ~150 kDa and imaged by negative-staining ET, and two individual 17 nm nascent HDL particles (36 Å resolution) that has a molecular mass of 140–240 kDa (protein portion: 56–84 kDa) and imaged by cryoET [7].

The successfully obtained 3D image of an individual particle is depended upon their four improvements/innovations: i) improved cryo-electron microscopy (cryoEM) sample preparation and Electron microscopy (EM) operation conditions [7]; ii) an optimized NS (OpNS) protocol [8,9]; iii) a high-resolution and high contrast sample preparation protocol, cryo-positive-staining (cryoPS) [10]; iv) a robust tomography reconstruction method, Individual Particle Electron Tomography (IPET) [6]. The detail improvements are descripted below:

## Optimization of Cryo-Electron Tomography (cryoET) Technique for High-Resolution Imaging of small Protein under a Near Native State

The advantage of cryoET is that the “native” structure of a biological macromolecule can be examined from a series of tilting angles in a defined physiological buffer. CryoET can provide structural details of complex cellular organizations at low resolutions (3–5 nm) [11]. However, small proteins (MM < 200 kDa) are very challenging to image with cryoET. Ren et al. [12] have spent years optimizing cryoET protocols and operating conditions based on their previous high-resolution experience in obtaining the 3.7Å atomic structure of aquaporin 1 transmembrane protein by cryo-crystallography [12–14] and their pioneer knowledge in electron microscopy theory [15–18]. Improvement include: i) optimization of the cryoEM specimen preparation for producing large (>~5 um), super flat and thin (<~70nm) amorphous ice; ii) maximization of the illumination dose to match the target to the resolution, iii) stabilization of the cryo-holder during tilting. These improvements resulted in the successful imaging of the smallest protein structure by cryoET, for example, HDL (total molecular mass from 100kDa to ~220kDa) [7,9,19–22].

## Development of Theoptimizednegative-Staining (OpNS) Protocol for Eliminating the Rouleau Artifact of Lipoproteins

**Figure 1:**
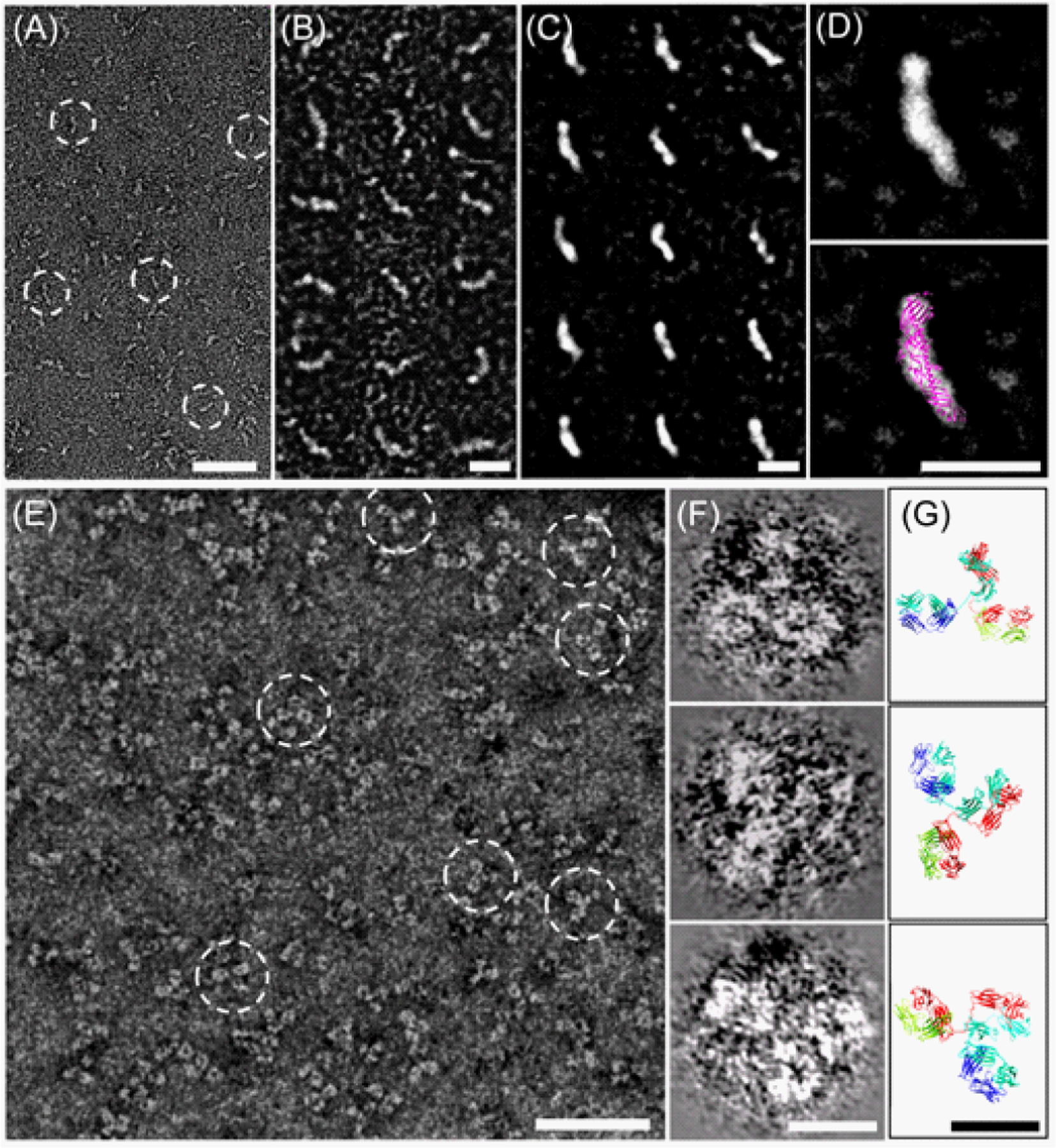
OpNS-EM images of CETP(10) and IgG antibody(6). OpNS can be used as a general protocol for non-lipoprotein imaging. One example is a structure-known small protein, 53kDa CETP. (A) OpNS images shows worm-shape of CETP, (B) selected raw particles and (C) reference-free class average displayed a near perfect fit to crystal structure in shape. Another example is a structural semi-known small protein, 160kDa IgG antibody, (E) OpNS images shows “Y-shape” structure of antibody. Selected raw particles (F) display three domain detail structure that are consistent to the crystal structure of antibody (G) (PDB 1igT), including the hole in domain. Scale bars: A and E, 50 nm; B-D, F, and G, 10 nm.

NS-EM, an easy, rapid, and qualitative method has been widely used for structural analysis of lipoprotein. However, conventional negative-staining protocol has the potential to introduce artifacts, such as rouleau formation of lipoprotein (particles are stocked together) [8,9]. Recently, Ren et al. [12] undertook studies of how the NS protocol affects to the rouleau artifact. As a result, we developed an OpNS EM protocol [8,9] that eliminates the rouleau artifact to visualize each individual lipoprotein particle [8,9].The OpNS images provide similar particle size and shape (difference <5%) as cryoEM, but provide higher contrast than cryoEM. The OpNS protocol represents a general method for examining lipoprotein species and complexes [8,9], such as nascent HDL, spherical HDL, plasma HDL, α-HDL [8], LDL, IDL and VLDL, LDL/CETP, LDL/CETP/HDL, LDL/antibody, HDL/LCAT and HDL/antibody [10]. Using the OpNS protocol, we have illustrated the CETP tunnel mechanism in cholesterol transfer among lipoprotein by examining over 300 sample conditions [10]. OpNS provides structural details of small proteins, such as 53 kDa CETP (Figure 1A–1D) and 160 kDa IgG antibody (Figure 1E–1F) that are difficult to be observed by other EM methods [6,10].

## Development of the Cryo-Positive-Staining (cryoPS) Protocol for High-Resolution Imaging of a Small Protein

Ren et al. [12] reported about the cryo-positive-staining protocol (cryoPS) [10], a combination of OpNS and conventional cryoEM. CryoP Scan provides detailed structure information of a single protein molecule that cannot be directly visualized by other techniques. For example, the cryoPS images of the 53 kDa CETP provides unprecedented secondary structure detail of an individual protein [10]. This protocol has been used to examine spherical HDL [8].The spherical HDL images also displayed fine details, such as a double helical liked-belt [8].The reason we named it cryo-PS is because the contrast of the image obtained with this method is inverted compared to the cryo-NS method reported previously [23]. In the cryo-NS protocol, an exceptionally high concentration of negative stain, 16% ammonium molybdate, is used to maintain the negative image contrast from cryo-NS specimens [23], whereas 1% uranylformate (UF) was used in the cryo-PS protocol. The cryo-PS-EM images show that the NS reagent, UF, penetrates the molecular surface, challenging the conventional wisdom that NS could only visualize the outer surface structure [24]. The mechanism of how UF penetrates the molecular surface is unknown. One possibility is binding of the uranylcation to available protein carboxyl groups, causing the surrounding (vitreous) negative stain to be of lower density than that of the protein+uranyl groups, thereby acting as a positive stain [10].

## Development of the Individual Particle Electron Tomography (IPET) for Determining a 3D Map of an Individual Protein

**Figure 2:**
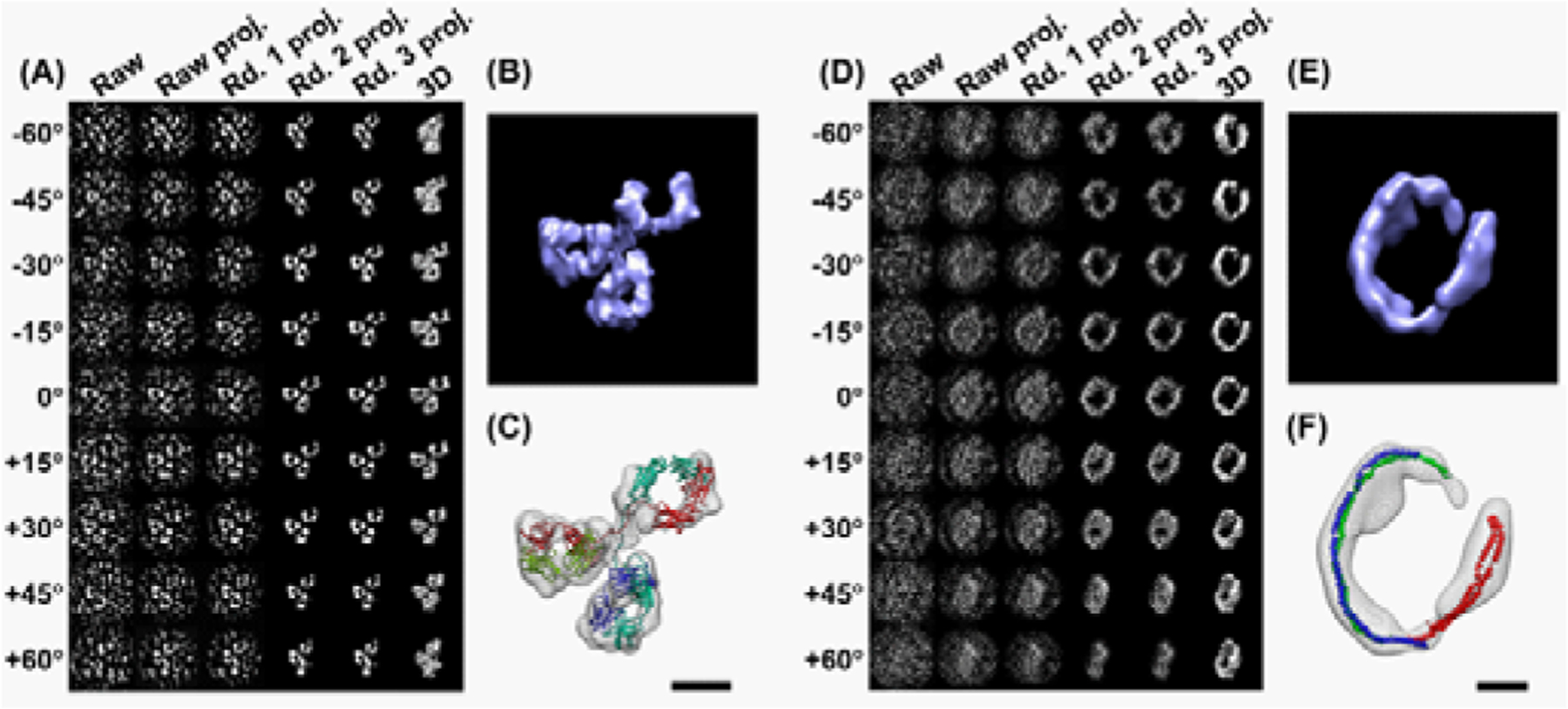
3D reconstructions of a IgG antibody by IPET (6). (A) A single IgG antibody was also imaged by OpNS tomography. By IPET, the images were also aligned, and (B) reconstructed into a 3D density map at resolution of ~14 Å. (C) Each domain of 3D map can near-perfect fit to the crystal structure domain in size and shape. (D) Using IPET, a 3D map of an individual 17nm HDL particle can be reconstructed. (E) 3D reconstruction at resolution of ~36 Å indicated a ring-shape structure of apoA-I. (F) The ring length suggested three apoA-Is within 17nm HDL. Scale bars, 5 nm.

IPET is a groundbreaking approach that allows determination of a high-resolution structure for a single molecule (Figure 2) [6,25]. IPET is a robust strategy/approach that does not require the pre-given initial model class averaging of multiple molecules or an extended ordered lattice, but can tolerate small tilt-errors for high-resolution single “snapshot” molecule structure determination. Thus, FETR/IPET provides a completely new opportunity for individual-molecule structure determination, and can be used to study the heterogeneous protein structure, dynamic character and equilibrium fluctuation of HDL and monomeric apoA-I [6].

In IPET reconstruction, each particle from a tilt series of micrographs is tracked and boxed, and then reconstructed into a 3D density map via a local refinement iteration reconstruction algorithm that involves a series of dynamic filters and automatically generated masks. To test the capability of IPET, we reconstructed an individual-molecule structure from a set of computational simulated cryoET images [7]. The reconstructed map shows detail structure of a single-molecule, suggesting the IPET has a potential to determine the secondary structure of an individual protein [6].

To validate this new approach, we applied this method to determine the real experimental structure of asymmetric small proteins, the 160 kDa IgG antibody respectively [6]. Step-by-step procedures show the OpNS image of a targeted IgG antibody images were graduated aligned together (Figure 2A), and reconstructed into a 3D density map at resolution of 14 Å (Figure 2B). By inserting the IgG domains from the crystal structure into each domain of the 3D density map, a near perfect fit in size and shape results (Figure 2C), suggesting the IPET is a power and reliable approach for single protein structure determination.

To validate the IPET capability on low-contrast cryoET images, we determined two 3D maps from two individual 17 nm nascent HDL particles [6]. As one example shown in Figure 2D, although cryoET images have much low contrast than that of OpNS, considering HDL has a total molecular mass of 120–220kDa (the protein moiety is only 74kDa), it is impressive that IPET can pick-up the signal from the noise images and reconstruct the signal into a 3D density map at a resolution of 36–42 Å (Figure 2E and 2F) [6]. Although the resolution is low compared to the conventional cryoEM approach in which a 3D map is reconstructed from an average of hundreds to thousands particles, considering the 3D map from IPET reconstructed from an individual protein, it is striking that an IPET 3D map contains structural information that is enough to answer important biological questions, such as the discoidal shape of 17 nm HDL, and the dynamics of the IgG domains [6].

Two commonly expressed limitations about our tomography reconstruction are missing-wedge and dose-limitation, issues that we discussed in detail in the IPET paper [6]. Briefly, regarding the missing-wedge issue, Ren et al. [12] suggested the missing wedge effect on a 3D reconstruction of a small and thin object, such as a protein, is limited. Indirectly evidence for our argument is that tomography 3D reconstruction also contains “wedge-shaped” missing-data (~22% of total data missing), due to a limited tilt range of +70° to −70° in most cases [6]; in comparison, in 2D electron crystallography there is a “cone-shape” missing-data (~18% of total data missing), at the tilted angle of 60° [12–14]. How come these 4% more missing-data affect the map and drop down its resolution from atomic level to below 20 Å? We believe that an in accurate alignment of tilt images is the key limitation to reconstruction quality rather than the “missing wedge” effect. To quote the discussion in our IPET paper [6], the 20 Å resolution limitation of cryoET is commonly quoted based on a theoretical calculation by Henderson [26]. However, one should notice two key parametric assumptions used in this calculation: the total dose of 5–20 e^−^/Å ^2^ and the X-ray solvent contrast factor of 0.28. First, in our experiments, total dose (~140 e^−^/Å ^2^) is much high [2,7]. Secondly, in our calculations, the electron scattering factor that has been collected in the *International Table for Crystallography* is significant higher than that of the x-ray scattering factor [15,17,18], resulting in a solvent contrast factor higher than 0.28. These two parameters result in a better resolution limitation than 20 Å.

In short, IPET is novel approach to achieve the 3D reconstruction from each individually targeted molecule instance, in which IPET went against the conventional wisdom that a single protein can NOT be reconstructed by EM. IPET achieved 3D reconstruction is from each individually targeted molecule instance that is free of conformational dynamics and heterogeneity, thus, IPET 3D structure can be treated as a snapshot of the dynamic structure of the macromolecule. By comparing these “snapshot” structures, this method could allow the study of macromolecular structural dynamics [21,27,28].

As a summary, the success visualization and reconstruction of the structure of these small proteins suggested that Ren et al. [12] novel EM techniques, including OpNS-EM, cryo-PS-EM, and IPET opens a door for the study of protein dynamics [6].
